# Effects of Applied Ratio of Nitrogen on the Light Environment in the Canopy and Growth, Development and Yield of Wheat When Intercropped

**DOI:** 10.3389/fpls.2021.719850

**Published:** 2021-08-19

**Authors:** Chaosheng Luo, Zengpeng Guo, Jingxiu Xiao, Kun Dong, Yan Dong

**Affiliations:** ^1^College of Resources and Environment, Yunnan Agricultural University, Kunming, China; ^2^School of Life Sciences, Lanzhou University, Lanzhou, China; ^3^College of Animal Science and Technology, Yunnan Agricultural University, Kunming, China

**Keywords:** intercropping, nitrogen level, wheat, light environment, yield, yield components

## Abstract

Changes in the light environment have an important effect on crop growth and yield. To clarify the effects of intercropping and the application of nitrogen on the yield of wheat and light within the crop canopy, the relationship between light and yield and their response to nitrogen fertilizer were studied. In a 2-year field experiment, the characteristics of growth, light, biomass, and yield of wheat were measured using three cropping arrangements (monocropped wheat, monocropped faba beans, and intercropped wheat/faba beans) and four levels of applied nitrogen, in groups termed N_0_ (0 kg/ha), N_1_ (90 kg/ha), N_2_ (180 kg/ha), and N_3_ (270 kg/ha). The results demonstrated that the application of nitrogen fertilizer increased wheat plant height, spike leaf length and width, and the number of leaves while significantly decreasing wheat canopy light transmittance (LT) and canopy photosynthetic active radiation transmittance (PART), by 7.5–71.1 and 12.7–75.1%, respectively. There was a significantly increased canopy photosynthetic active radiation interception rate (IPAR) of 7.5–97.8% and an increase in biomass of 9.6–38.4%, of which IPAR, biomass, and yield were highest at the N_2_ level. Compared with monocropping, intercropping increased parameters of wheat growth to varying degrees. Intercropping decreased LT and PART by 10.8–46.4 and 15.7–58.7%, respectively, but increased IPAR by 0.1–66.0%, wheat biomass and yield by 7.5–17.4 and 27.7–47.2%, respectively. The mean yield of intercropped wheat increased by 35.8% over 2 years, while the mean land equivalent ratio (LER) was 1.36, for which a values greater than 1 indicates that wheat and faba bean intercropping is advantageous. Correlation analysis demonstrated that there was a very significant negative correlation between wheat LT and yield, while simultaneously demonstrating a very significant positive correlation between PART and IPAR with yield, indicating that the efficient interception and utilization of light energy in intercropping was the basis for the higher biomass and yield of wheat. In summary, wheat/faba bean intercropping and the application of nitrogen at 180 kg/ha were effective in increasing wheat yield.

## Introduction

Wheat (*Triticum aestivum* L.) is the second-largest food crop after rice and is widely cultivated and produced around the world. China is one of the major wheat-producing countries, having an area of cultivation and total yield that ranks first of any country around the globe, and mean yield per unit area ranking tenth (The Food and Agriculture Organization Corporate Statistical Database (FAOSTAT), 2020). As the global population continues to rise, the demand for wheat is expected to grow at an annual rate of 1.6% by 2050 (Singh et al., 2016). Therefore, it is important that the yield of wheat is carefully considered so that the requirements of humans are fulfilled and their nutritional balance maintained. Light energy is an important power resource for crop physiological function (respiration, photosynthesis, and transpiration). The rate of interception and efficiency of utilization in a planted crop play major roles in determining the final yield (Wang et al., 2015; Raza et al., 2019; Gong et al., 2020).

Nitrogen is an essential nutrient for crops, and its application can be effective for increasing crop yield (Guo et al., 2019). The quantity of nitrogen fertilizer clearly influences the structure of the crop population, the rate of light energy interception by the canopy, the crop seed setting rate, 1000-grain weight, and yield (Li D. Q. et al., 2010). A large number of previous studies have demonstrated that application of nitrogen can increase the height of the crop, numbers of leaves, leaf area index, the crop canopy photosynthetic active radiation interception rate, and rate of photosynthesis, all beneficial for crop growth and yield (Zhang et al., 2018). On the other hand, excessive use of nitrogen fertilizer has been shown to result in light to of the middle and lower parts of the crop canopy being blocked, resulting in a reduction in light energy conversion efficiency (Tang et al., 2012). Additionally, a reduction in light transmittance through the crop population reduces light in the lower parts of the canopy, accelerating senescence of the lower leaves, thereby affecting crop yield (Li et al., 2011). For example, Yang et al. (2017) found that the application of nitrogen fertilizer significantly reduced light transmittance in rice crops, causing leaves in the middle to not receive sufficient sunlight for photosynthesis, affecting rice yield to a certain extent.

An appropriate intercropping regimen can be effective in improving the structure of the crop population, making full use of light and heat resources, maximizing the advantages of intercropping, thereby achieving optimal yield (Willey, 1979; Hu et al., 2016). A study by Xu et al. (2010) indicated that the increased yield of maize intercropped with pepper was due to the significant increase in canopy interception of photosynthetically active radiation and the rate of utilization. A study by Liu et al. (2017) and Raza et al. (2019) demonstrated that intercropping changed the light conditions in maize, improving photosynthesis in the leaves, increasing yield, and demonstrating the advantages of intercropping soybean with maize. Effective interception and utilization of light energy through the use of intercropping represents the basis for achieving high yields (Wang et al., 2015).

Wheat and faba bean intercropping is a typical planting arrangement in southwest China. Studies have demonstrated that wheat and faba bean intercropping provides clearly superior yields, differences that are affected by levels of nitrogen (Zhu et al., 2017). Over recent years, previous studies of wheat and faba bean intercropping have focused principally on the effect of intercropping and the nutritional and ecological mechanisms of disease control (Zhu et al., 2017; Ma et al., 2019), in addition to interspecies competition and efficient utilization of resources (Bai et al., 2018). However, few studies have been published in which the advantages and photosynthetic mechanisms of increases in crop yield in such intercropping systems have been investigated, especially the influence of intercropping on wheat yield and canopy light levels for different levels of applied nitrogen. Therefore, the present study investigated a wheat and faba bean intercropping model with varying levels of applied nitrogen, the influence on light transmittance through the wheat canopy, the photosynthetically active radiation interception rate, and yield. The relationship between change in canopy light levels and yield, and the response to nitrogen fertilizer application in an intercropped system were analyzed, and the photosynthetic mechanism in intercropping on wheat yield additionally elucidated. The scientific basis was established for an intercropping system with a high grain yield and efficient utilization of resources, and also establishing theoretical guidance for the most appropriate nitrogen nutrient management for intercropping systems.

## Materials and Methods

### Experimental Site and Cultivars

The 2-year field experiments were conducted in 2016–2017 and 2017–2018 in E Feng village (102°24′E, 24°11′N, altitude: 1691 m), in E Shan County in Yuxi, Southern Yunnan Province, China. The region has a mid-subtropical monsoon climate with an average annual temperature and rainfall of 16.3°C and 1120 mm, respectively. The meteorological data during the crop’s growing season are presented in Table 1. The crop formerly grown on the plots was leek, the soil was a paddy type, with 28.9 g/kg organic matter in the top 20 cm of topsoil, total nitrogen: 2.2 g/kg, total phosphorus: 0.8 g/kg, total potassium: 18.3 g/kg, alkali-hydrolyzable nitrogen: 102.0 mg/kg, available phosphorus: 36.9 mg/kg, and available potassium: 100.5 mg/kg, with a pH of 6.8.

**TABLE 1:** Mean monthly temperatures and rainfall during the two planting seasons in 2016–2017 and 2017–2018.

Month		October	November	December	January	February	March	April
Temperature (°C)	2016–2017	19.2	14.3	11.8	11.9	12.6	14.6	17.4
	2017–2018	17.1	13.1	9.8	9.6	10.4	14.6	17.4
Rainfall (mm)	2016–2017	117.8	74.3	13.4	18.9	25.8	30.3	45.2
	2017–2018	135.9	34.7	1.7	35.8	0.4	40.9	21.5

The test varieties *Vicia faba* L. cv. ‘Yuxi dalidou’ and *Triticum aestivum* L. cv. ‘Yunmai-52’ were investigated in the present study. Both cultivars were purchased from the Institute of Food Crops, Yunnan Academy of Agricultural Sciences.

### Experimental Design and Crop Management

**FIGURE 1:**
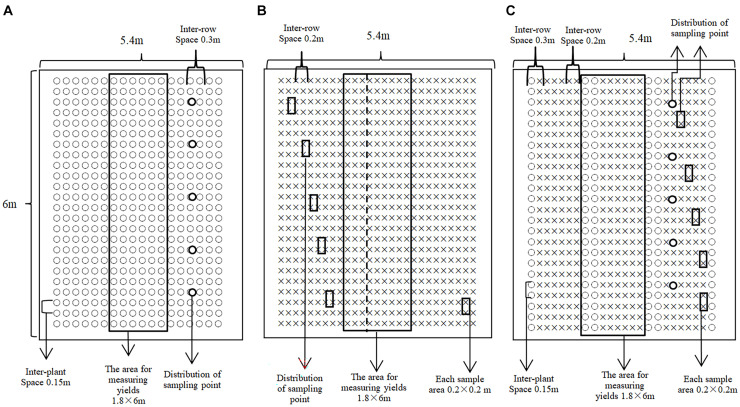
Plot diagram of planting pattern in field plot experiment: **(A)** plot map of monocropped faba bean plot; **(B)** plot map of monocropped wheat plot; and **(C)** plot map of intercropped faba bean/wheat (O represents faba bean and X represents wheat).

The study was designed around two factors (A and B), where factor A represented one of three planting arrangements: wheat monocropping (MW), faba bean monocropping (MF), and wheat with faba bean intercropping (W/F). Factor B represented one of four nitrogen application levels, namely, no nitrogen (N_0_), low nitrogen (N_1_), conventional levels of nitrogen (N_2_), and high levels of applied nitrogen (N_3_). The corresponding quantity of applied nitrogen to wheat was 0, 90, 180, and 270 kg/ha, respectively, double that applied to faba beans (i.e., 0, 45, 90, and 135 kg/ha). The complete treatment levels were divided into 12 treatments, with 3 repeats per treatment, for a total of 36 plots, using a completely randomized block design on plots that were 6.0 m long by 5.4 m wide (6.0 m × 5.4 m = 32.4 m^2^). Wheat was strip sown (row spacing: 0.2 m; quantity sown: 18 g/row), with monocropped wheat (MW) at 27 rows/plot, or intercropped wheat (IW) at 18 rows/plot, and faba bean planted on demand (row spacing: 0.3 m, plant spacing: 0.15 m), with monocropped faba beans at 18 rows/plot, and intercropped faba beans at 6 rows/plot. The intercropped plot was planted with two rows of faba beans and six rows of wheat, with three wheat and three faba bean planting belts in each intercropped plot (Figure 1).

The fertilizers tested were urea (N: 46%), superphosphate (P: 7%), and potassium sulfate (K: 41%). The phosphate and potassium fertilizers were both used at 90 kg/ha as base fertilizers, applied once by hand, prior to sowing on a single occasion. The faba bean nitrogen fertilizer was applied as a base fertilizer once prior to sowing. For the wheat, the nitrogen fertilizer was applied twice as a base fertilizer and also as a topdressing fertilizer (in 50% proportions), that is, half was applied prior to sowing and half at the wheat jointing stage in water. The daily management of other agronomic field measures (irrigation and weeding, etc.) was consistent with local practice. The wheat and faba beans were sown on October 21, 2016 and October 23, 2017, while the wheat was harvested on April 19, 2017 and April 25, 2018.

### Determination of Plant Characteristics and Methods

#### Determination of Wheat Plant Height, Spike Leaf Length, Spike Leaf Width, and Numbers of Leaves

At the grain filling stage of the wheat (approximately 95 days after sowing), 10 wheat plants were selected in each plot using an “S” type selection method. The height above ground, and flag leaf length and width of the wheat were measured using a steel tape measure (accuracy<0.1 cm). In addition, the number of leaves tillers in each selected sample was counted.

#### Determination of Light Levels Within the Wheat Canopy

The canopy light intensity (Lux) levels and photosynthetically active radiation (in μmol m^–2^ s^–1^) were measured in clear and cloudless conditions at the wheat grain filling stage, using a GLZ-C-G photosynthetically active radiation meter (Zhejiang Topu Yunnong Science and Technology Company). Measurements were obtained in accordance with field microclimate measurement standards (Guo et al., 2019) in both monocropped and IW (rows of wheat adjacent to faba beans were selected). Light levels were measured 0.2 m above ground (lower level), at 2/3 plant height (mid-level), and at the crown (upper part) during the period from 12:00 noon to 14:00, at the wheat filling stage. From these values, canopy light transmittance (LT), canopy photosynthetically active radiation transmittance (PART), and canopy photosynthetically active radiation interception rate (IPAR) were calculated, as follows (Serrano et al., 2000):

LT (%) = light intensity at the measurement site within the canopy / light intensity at the top of the canopy × 100%.

PART (%) = photosynthetically active radiation at the measurement site within the canopy / photosynthetically active radiation at the top of the canopy × 100%.

IPAR (%) = (photosynthetically active radiation value at the top of the canopy − photosynthetically active radiation value at the measurement site) / canopy top photosynthetic active radiation value × 100%.

#### Determination of Wheat Biomass

For the measurement of wheat growth parameters, five sampling areas were selected from each plot, in accordance with a pre-determined five-point sampling method, the size of which was 0.2 m × 0.2 m (Figure 1). For this, the parts of the plants above the ground (stem, leaves, and spikes) of both monocropped and IW were collected, and incubated at 105°C for 15 min, then dried to a constant weight at 65°C, prior to weighing and calculation of the aboveground biomass per unit area.

#### Determination of Wheat Yield and Yield Components

At maturity, after removing the three side rows in each yield measurement plot, six successive rows of wheat were evenly cut, and the thousand kernel weight and grain yield of wheat were measured following air-drying. Ten plants were randomly selected from each plot to determine the spike length, spike weight, spike number, and numbers of grains per spike. The middle six rows of faba beans were harvested from the monocropping plot. A complete band was harvested from the intercropped plot in the middle, including two rows of faba beans and six rows of wheat (Figure 1). Finally, the dominant land equivalent ratio (LER) for intercropping was calculated, as follows (Willey, 2007):

$$\text{LER}=Y_{\text{iw}}/Y_{\text{mw}}+Y_{\text{if}}/Y_{\text{mf}}$$

where, *Y*_iw_ and *Y*_if_ represent total wheat and faba bean yield in the intercropped yield measurement area, respectively; *Y*_mw_ and *Y*_mf_ represent total wheat and faba bean yield in the monocropped yield measurement area, respectively; LER > 1, indicated that intercropping was advantageous, and disadvantageous where LER < 1.

### Statistical Analysis

Microsoft Excel 2010 software was used to collate and analyze the data. SPSS, 2008 v21.0 software (SPSS Inc., Chicago, IL, United States) was used to calculate two-way ANOVAs and for correlation analysis. A least significant difference (LSD) method was used for multiple comparisons. All differences were considered significant at *P* < 0.05.

## Results and Analysis

### Effects of Application of N and Intercropping on the Growth Characteristics of Wheat

The application of nitrogen increased growth parameters (plant height, number of leaves, and spike leaf length and width) of monocropped and IW for 2 years, the lowest level of which was N_0_ while the highest was N_3_ (Table 2). Compared with N_0_, the height of wheat in the 2-year monocropped and intercropped treatments increased significantly by 14.5–38.1% at the N_1_–N_3_ levels, although the difference at the N_1_ level in 2017–2018 was not significant. The number of MW leaves increased by 85.6% due to the N_3_ treatment in 2017–2018, while intercropping resulted in a significant increase of 21.7–30.2% at the N_3_ level. MW spike leaf length increased significantly by 12.3–17.8% at each N level, and spike leaf width increased significantly only at the N_2_ and N_3_ levels, by 21.1–33.3%, while leaf length in IW increased significantly only at the N_2_ and N_3_ levels, by 23.2–38.9%, with no significant difference in spike leaf width. The results demonstrate that the application of nitrogen was beneficial for the growth of wheat, especially at the highest nitrogen (N_3_) level, while the response of wheat plant height to nitrogen was greater than that of spike leaf length, spike leaf width, or numbers of leaves.

**TABLE 2:** Effects of applied nitrogen and intercropping on wheat growth parameters in the mean of two growing seasons.

Year	N levels	Plant height (cm)		Leaves number		Spike leaf length (cm)		Spike leaf width (cm)	
		MW	IW	MW	IW	MW	IW	MW	IW
2016–2017	N0	94.0 ± 4.0b	97.4 ± 3.7b	44.7 ± 5.6a	49.3 ± 7.1b	19.5 ± 3.3c	21.1 ± 2.9b	1.9 ± 0.2b	2.0 ± 0.2a
	N1	97.8 ± 3.1b	102.1 ± 6.6b	47.1 ± 2.8a	50.7 ± 5.3ab	21.9 ± 2.3b	23.6 ± 2.3ab	2.0 ± 0.2ab	2.1 ± 0.4a
	N2	107.6 ± 5.6a	112.0 ± 4.1a	53.7 ± 4.2a	53.8 ± 2.0ab	22.9 ± 1.3b	26.0 ± 2.4a*	2.3 ± 0.1a	2.4 ± 0.1a
	N3	110.9 ± 2.2a	112.4 ± 3.0a	53.4 ± 7.4a	60.0 ± 4.4a	26.8 ± 1.6a	27.5 ± 1.1a	2.4 ± 0.0a	2.4 ± 0.3a
	Mean	102.6	106.0	49.7	53.4	23.4	24.2	2.2	2.3
2017–2018	N0	78.0 ± 5.6d	86.4 ± 2.0d*	15.3 ± 3.2b	25.5 ± 3.8b*	18.0 ± 0.8c	23.6 ± 3.7b*	1.8 ± 0.3b	2.0 ± 0.2a
	N1	88.3 ± 5.5c	94.3 ± 0.9c	20.0 ± 1.6b	26.1 ± 4.1b*	24.2 ± 1.8b	26.9 ± 2.8ab	2.2 ± 0.1ab	2.3 ± 0.1a
	N2	99.9 ± 5.0b	100.0 ± 3.8b	24.1 ± 3.9ab	30.7 ± 2.2ab*	27.1 ± 0.3ab	30.4 ± 2.5a	2.4 ± 0.2a	2.4 ± 0.2a
	N3	110.1 ± 3.6a	107.7 ± 1.5a	31.4 ± 7.4a	33.2 ± 2.6a	28.8 ± 1.8a	32.8 ± 1.3a*	2.3 ± 0.1a	2.3 ± 0.2a
	Mean	94.1	97.1	22.8	28.9	24.5	28.4	2.2	2.2

*MW, monocropped wheat; IW, intercropped wheat. Different small letters represent significant differences between nitrogen application rates for monocropping and intercropping, respectively, at the 0.05 level. Asterisk represents significant differences between monocropping and intercropping for the same level of nitrogen application at the 0.05 level. Mean: average nitrogen levels for the same planting arrangement.*

Compared with monocropping, IW plant height, numbers of leaves, leaf length, and leaf width increased by 3.5, 7.6, 8.0, and 3.6% at the N_0_–N_3_ levels in 2016–2017, while only spike leaf length at the N_2_ level increased significantly. In 2017–2018, the height of wheat increased significantly, by 10.8% at the N_0_ level, and by a mean of 5.0% at the N_1_–N_3_ level. The number of leaves on IW increased by 27.4–66.6% at the N_0_–N_2_ levels, while wheat leaf width increased significantly at the N_0_–N_3_ levels. However, there was no significant increase in leaf width in IW (Table 2). The results demonstrate that intercropping increased wheat plant height, leaf number, and spike leaf length, but the advantage of intercropping decreased with increasing levels of nitrogen.

### Effects of Applied N and Intercropping on the Light Environment Within the Wheat Canopy

#### Effect on Light Transmittance

**FIGURE 2:**
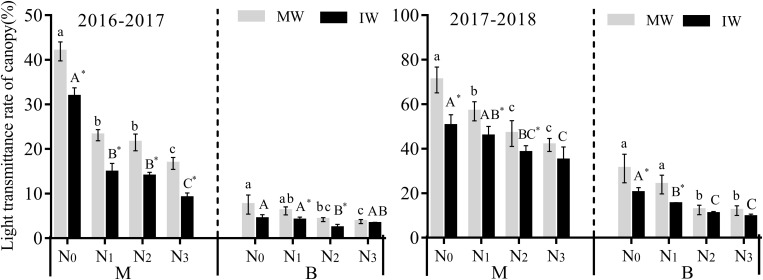
Effects of applied nitrogen and intercropping on light transmittance of wheat canopy. MW, monocropped wheat; IW, intercropped wheat; M, middle of canopy; B, below of canopy. The different capital letters and small letters represent significant differences between different N levels in the same planting pattern at *P* < 0.05 levels. * Represents significant difference between monocropped and intercropped patterns at the same N level (*P* < 0.05).

Canopy light transmittance in the middle and lower parts of the canopy for monocropped and IW over 2 yearsdecreased with increasing nitrogen level, in the order N_0_ > N_1_ > N_2_ > N_3_. LT in the vertical direction for the middle part of the canopy was greater than the lower part (Figure 2). Compared with N_0_, LT in the middle of the canopy of MW decreased significantly by 19.9–59.9% for the 2 years at the N_1_∼N_3_ level, while that of IW at the N_2_ and N_3_ levels decreased significantly by 24.2–71.7%. The decrease was significant only at the N_1_ level in 2016–2017, at 44.9%. LT in the lower canopy of MW decreased significantly by 44.0–61.4% at the N_2_ and N_3_ levels, and decreased significantly in 2016–2017 only at the N_2_ level, by 46.5%, while that of IW decreased significantly by 24.8–53.5% at the N_1_–N_3_ levels in 2017–2018. The results demonstrate that nitrogen significantly affected LT in the wheat canopy, especially at high levels of nitrogen (N_3_). In addition, the influence on LT of nitrogen in the middle part of the wheat canopy was greater than that in the lower part.

Compared with monocropping, LT in the middle of the canopy of wheat decreased significantly by 24.1–46.3% at the N_0_–N_3_ level in 2016–2017, while LT in the lower part of the canopy decreased significantly by 34.4–45.2% only at the N_1_ and N_2_ levels. In 2017–2018, LT in the middle part of the canopy decreased significantly by 18.4–28.9% for the N_0_∼N_2_ levels, while LT within the lower canopy decreased significantly by 35.0–36.4% only at the N_0_ and N_1_ levels, although there was no significant difference at medium and high nitrogen levels (N_2_ and N_3_) (Figure 2). The results demonstrated that LT within the canopy of IW was lower than that in monocropping, the difference significant at low nitrogen levels, with no significant differences at middle and high nitrogen levels.

#### Effect on Photosynthetically Active Radiation Transmittance

**FIGURE 3:**
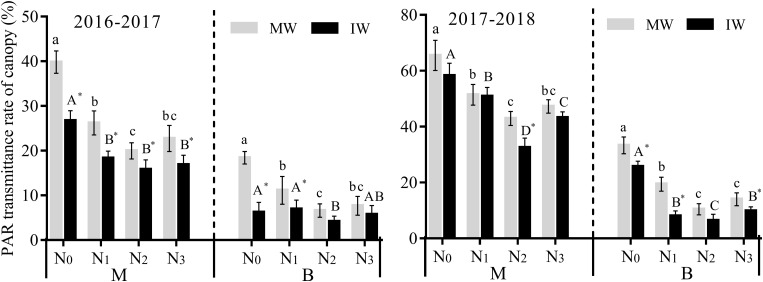
Effects of applied nitrogen and intercropping on photosynthetically active radiation transmittance (PART) in the wheat canopy. MW, monocropped wheat; IW, intercropped wheat; M, middle of canopy; B, below of canopy. The different capital letters and small letters represent significant differences between different N levels in the same planting pattern at *P* < 0.05 levels. * Represents significant difference between monocropped and intercropped patterns at the same N level (*P* < 0.05).

It can be seen from Figure 3 that in both years of the study, PART in the middle and lower parts of the canopy in monocropped and IW decreased initially then increased for increasing levels of nitrogen, indicating that N_0_ > N_1_ > N_3_ > N_2_, and that changes in PART in the middle part of the canopy > the lower part. Compared with N_0_, PART in the middle of the canopy of monocropped and IW over 2 years significantly decreased by 21.5–49.8 and 12.7–44.2% at the N_1_–N_3_ levels, while PART in the lower canopy of monocropped and IW decreased significantly by 39.5–68.7 and 33.5–75.1% at the N_1_–N_3_ levels, except for the N_1_ level in 2016–2017.

Compared with monocropping, PART in the middle of the wheat canopy decreased with increasing nitrogen over the 2 years. For the N_0_–N_3_ levels in 2016–2017 it decreased significantly by 20.7–32.8%, while in 2017–2018, the decrease was significant only for the N_2_ level, by 24.2%. PART in the lower part of the wheat canopy was significantly lower for the N_0_ and N_1_ levels, by 22.8–58.7%, while the decrease was significant only at the N_3_ level in 2017–2018, a decrease of 30.0%. The results demonstrate that intercropping reduced PART in the wheat canopy and improved light levels.

#### Effect on Photosynthetically Active Radiation Interception Rate

**FIGURE 4:**
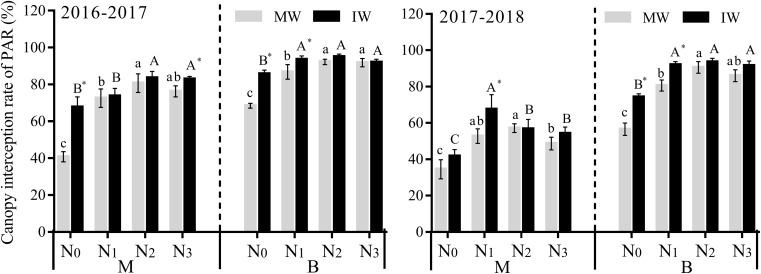
Effects of applied nitrogen and intercropping on photosynthetically active radiation transmittance (IPAR) in the wheat canopy. MW, monocropped wheat; IW, intercropped wheat; M, middle of canopy; B, below of canopy. The different capital letters and small letters represent significant differences between different N levels in the same planting pattern at *P* < 0.05 levels. * Represents significant difference between monocropped and intercropped patterns at the same N level (*P* < 0.05).

As displayed in Figure 4, compared with N_0_, the IPAR in the middle of the canopy in monocropped and IW for the 2 years increased significantly by 40.9–97.8 and 9.0–61.5% at the N_1_–N_3_ level, respectively, with a mean increase of 70.3–30.3%, while IPAR in the lower canopy of monocropped and IW increased significantly by 26.9–61.2 and 7.5–26.2% at the N_1_–N_3_ levels, respectively, with a mean increase of 42.2–16.8%. Compared with monocropping, the IPAR in the middle and lower part of the wheat canopy increased by 0.2–66.0 and 0.3–31.5 at the N_0_–N_3_ level, respectively, with a mean increase of 17.6–11.5%, while the lower part of the canopy displayed a significant difference only at the N_0_ and N_1_ levels for the 2 years. The results demonstrate that conventional nitrogen intercropping increased IPAR within the wheat canopy and improved its light levels.

As shown in Table 3, the nitrogen levels and planting pattern had significant influence over the 2 years on LT, PART, and IPAR in the middle and lower parts of the wheat canopy, as demonstrated by ANOVA. Additionally, the interaction of the two factors significantly affected PART and IPAR in the middle and lower parts of the wheat canopy.

**TABLE 3:** Variance analysis of applied nitrogen and intercropping on canopy light environment and yield of wheat.

Year	Factor	Middle canopy			Below canopy			Yield
		CT	PART	IPAR	CT	PART	IPAR	
2016–2017	N	248.6**	34.2**	18.1**	21.9**	29.7**	75.3**	17.9**
	CP	160.6**	12.7**	6.6*	10.0**	33.2**	46.3**	34.9**
	N *×* CP	0.8^ns^	9.4*	4.9*	1.0^ns^	6.7**	5.6**	0.7^ns^
2017–2018	N	25.9**	61.1**	58.9**	31.5**	48.3**	49.4**	29.1**
	CP	36.3**	45.2**	33.1**	23.2**	35.8**	99.6**	67.4**
	N × CP	2.4^ns^	21.8**	13.0**	3.2^ns^	18.9**	39.9**	0.6^ns^

N, N level; CP, cropping pattern. *P < 0.5; **P < 0.01; ns: not significant.

### Effects of Applied N and Intercropping on Wheat Biomass at the Filling Stage

**FIGURE 5:**
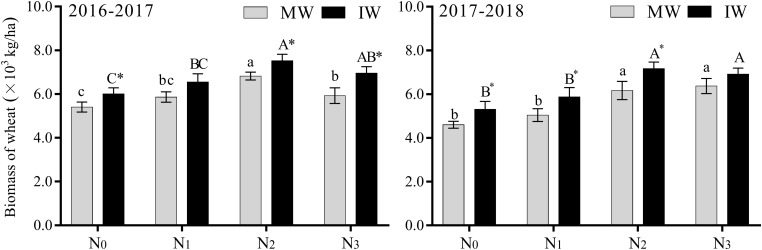
Effects of applied nitrogen and intercropping on wheat biomass at the filling stage. MW, monocropped wheat; IW, intercropped wheat; M, middle of canopy; B, below of canopy. The different capital letters and small letters represent significant differences between different N levels in the same planting pattern at *P* < 0.05 levels. * Represents significant difference between monocropped and intercropped patterns at the same N level (*P* < 0.05).

As displayed in Figure 5, the biomass of monocropped and IW increased initially, then decreased with increasing levels of nitrogen, reaching its maximum at the N_2_ level. Compared with N_0_, the biomass of monocropped and IW increased significantly by 9.6–38.4 and 15.8–35.1%, respectively, at the N_2_ and N_3_ levels, while the level increased at N_1_, but not significantly. Compared with monocropping, the biomass of 2-year IW increased with increasing nitrogen level, for which N_0_ and N_2_ intercropping increased significantly, by 10.4–16.2%, while the increase was significant at the N_3_ level only in 2016–2017, by 17.4%. There was no significant increase due to intercropping at the N_1_ level.

### Effects of Applied N and Intercropping on Wheat Yield and Its Components

From Table 4, compared with N_0_, spike length and weight, grain number per spike, numbers of spikes, and 1000-grain weight of the 2-year monocropped and IW increased by 5.9–34.3, 0.1–19.2, 0.3–19.7, 18.4–90.4, and 2.0–8.4%, respectively at the N_1_–N_3_ levels. The numbers of spikes and grain number per spike increased significantly at the N_1_–N_3_ levels, and spike length increased significantly at the N_2_ and N_3_ levels. Panicle weight displayed annual and seasonal changes over the 2 years, while 1000-grain weight increased significantly only at the N_2_ and N_3_ levels, and there was no significant increase for different nitrogen application levels in MW. Compared with monocropping, spike weight, numbers of spikes, grain number per spike, and 1000-grain weight of wheat at different nitrogen levels increased by 1.7–45.7, 0.7–22.9, 4.3–32.0, and 0.1–5.0%, respectively for the N_0_-N_3_ levels, but the number of spikes and grains per spike increased significantly only at the N_3_ level.

**TABLE 4:** Effects of applied nitrogen and intercropping on yield components of wheat.

Year	N level	Spike length (cm)		Spike weight (g)		Spike number		Grain number per spike		1000-grain weight (g)	
		MW	IW	MW	IW	MW	IW	MW	IW	MW	IW
A	N0	9.8 ± 0.7b	10.2 ± 0.3b	5.2 ± 0.2a	5.9 ± 0.5a	6.8 ± 0.4c	8.7 ± 2.1c	63.7 ± 3.9b	66.5 ± 6.5b	58.9 ± 1.3a	59.6 ± 2.5b
	N1	10.7 ± 0.4b	10.8 ± 0.1b	5.6 ± 1.3a	5.9 ± 0.8a	8.9 ± 1.2b	10.3 ± 0.3b	74.4 ± 5.6a	75.6 ± 5.1ab	60.1 ± 1.8a	62.1 ± 1.9ab
	N2	12.9 ± 0.1a	13.7 ± 0.6a	6.2 ± 0.4a	6.5 ± 0.8a	9.7 ± 0.8	12.8 ± 0.4a*	70.6 ± 4.3ab	79.6 ± 3.6a*	62.2 ± 2.9a	64.6 ± 1.2a
	N3	13.0 ± 0.7a	13.3 ± 0.3a	6.0 ± 0.4a	6.1 ± 0.3a	11.9 ± 0.3a	13.1 ± 0.2a	63.9 ± 5.4b	67.6 ± 2.2b	60.3 ± 0.8a	63.3 ± 0.8a*
B	N0	7.2 ± 0.3c	7.0 ± 0.5b	2.3 ± 0.3b	2.6 ± 0.2b	7.3 ± 0.9c	9.7 ± 1.8c	31.5 ± 2.8c	38.7 ± 5.4c	60.8 ± 4.9b	62.8 ± 3.5b
	N1	9.3 ± 1.2b	10.1 ± 0.2ab	3.5 ± 0.7b	5.1 ± 0.4a*	9.9 ± 0.3b	12.8 ± 0.3b*	48.3 ± 7.8b	59.1 ± 2.1b*	63.7 ± 1.8ab	65.4 ± 4.1ab
	N2	11.4 ± 0.9a	11.0 ± 0.8a	5.2 ± 0.7a	5.1 ± 0.7a	10.7 ± 0.8	12.8 ± 0.4a*	73.6 ± 7.4a	70.9 ± 8.9a	69.3 ± 5.4a	69.3 ± 6.0a
	N3	12.2 ± 0.7a	11.7 ± 0.5a	5.2 ± 0.6a	4.9 ± 0.4a	13.9 ± 0.3a	14.5 ± 0.4a	70.9 ± 6.0a	71.4 ± 6.4a	65.9 ± 0.7ab	68.3 ± 2.7a

*A, 2016–2017; B, 2017–2018. Different small letters represent significant differences between nitrogen application rates for monocropping and intercropping, respectively, at the 0.05 level. Asterisk represents significant differences between monocropping and intercropping for the same level of nitrogen application at the 0.05 level. Mean: average nitrogen levels for the same planting arrangement.*

As displayed in Table 5, the yield of monocropped and IW was least at the N_0_ level and highest at the N_2_ level. Compared with N_0_, the wheat yield of IW increased significantly by 6.5–26.2 and 6.3–27.0% at the N_1_–N_3_ level for the 2 years, while there was no significant increase in the yield of MW at each nitrogen level. Compared with monocropping, 2-year intercropping significantly increased wheat yield at all nitrogen levels (Table 4), while the percentage increase of intercropped yield decreased with increasing nitrogen levels. Nitrogen levels and the pattern of planting significantly affected yield, but their interaction had no significant effect on yield (Table 3). The LER is an important index that can establish whether there is an advantage to intercropping. Over the 2 years, mean LER for each nitrogen application level was 1.37 and 1.34, indicating that wheat and faba bean intercropping displayed a clear advantage in yield, but with increased applied nitrogen, the increase in yield due to intercropping gradually decreased.

**TABLE 5:** Effects of applied nitrogen and intercropping on wheat yield and LER.

N levels	2016–2017 (×10^3^ kg/ha)			2017–2018 (×10^3^ kg/ha)			LER	
	MW	IW	Increase (%)	MW	IW	Increase (%)	2016–2017	2017–2018
N_0_	3.33 ± 0.6a	4.89 ± 0.4b*	46.83	3.42 ± 0.4b	5.03 ± 0.2b*	47.20	1.42 ± 0.17	1.40 ± 0.09
N_1_	4.24 ± 0.5a	5.88 ± 0.3a*	38.61	4.56 ± 0.6a	6.02 ± 0.6a*	32.11	1.41 ± 0.09	1.31 ± 0.03
N_2_	4.67 ± 0.6a	6.17 ± 0.3a*	32.26	4.78 ± 0.4a	6.39 ± 0.3a*	33.82	1.34 ± 0.08	1.34 ± 0.04
N_3_	4.08 ± 0.3a	5.21 ± 0.5a*	27.66	4.18 ± 0.3ab	5.35 ± 0.3a*	28.00	1.32 ± 0.02	1.29 ± 0.03
Mean	4.08	5.54	36.34	4.23	5.70	35.28	1.37	1.34

*Different small letters represent significant differences between nitrogen application rates for monocropping and intercropping, respectively, at the 0.05 level. Asterisk represents significant differences between monocropping and intercropping for the same level of nitrogen application at the 0.05 level. Mean: average nitrogen levels for the same planting arrangement.*

### Correlation Analysis Between Canopy Light Levels and Yield, and Yield Components for Wheat

To clarify the relationship between wheat canopy light levels and wheat yield and yield factors, the correlation between wheat canopy LT, PART, IPAR, and wheat spike length, spike weight, spike number, grain number per spike, 1000-grain weight, and grain yield were analyzed (Table 6). The results demonstrate that LT in the middle and lower parts of the canopy was significantly negatively correlated with spike length, spike weight, spike number, 1000-grain weight, and yield (except spike weight in 2016–2017). PART in the middle and lower parts of the canopy was significantly negatively correlated with yield and yield factors (except numbers of grains per spike in 2016–2017 and 1000-grain weight in 2017–2018). IPAR in the middle and lower part of the canopy was significantly or very significantly positively correlated with yield and yield factors (except for spike length and spike weight in the middle of the canopy in 2016–2017, while there was no significant correlation with 1000-grain weight in 2017–2018). The results demonstrate that wheat yield was closely related to canopy LT, PART, and IPAR, a high IPAR being the prerequisite for a high yield of wheat.

**TABLE 6:** Correlation analysis between canopy light levels and yield, and the yield components of wheat.

Year	Item	Middle of canopy			Below of canopy		
		LT	PART	IPAR	LT	PART	IPAR
2016–2017	Spike length	−0.724**	−0.562*	0.325	−0.669**	−0.445**	0.701**
	Spike weight	−0.436*	−0.521**	0.238	−0.518*	−0.456*	0.503*
	Spike number	−0.323**	−0.412*	0.527**	−0.229*	−0.332**	0.601**
	Grain number per spike	–0.372	−0.425*	0.493*	0.244	0.212	0.531**
	1000-grain weight	−0.615**	−0.312*	0.490*	−0.536**	–0.425	0.620**
	Grain yield	−0.796**	−0.704**	0.676**	−0.796**	−0.496**	0.794**
2017–2018	Spike length	−0.735**	−0.812**	0.718**	−0.751**	−0.612**	0.51**
	Spike weight	−0.724**	−0.654**	0.745**	−0.767**	−0.457**	0.609**
	Spike number	−0.458**	−0.448**	0.662**	−0.714**	−0.412**	0.612**
	Grain number per spike	−0.761**	−0.792**	0.806*	−0.838**	−0.712**	0.643**
	1000-grain weight	0.068	–0.045	0.077	–0.038	−0.313*	–0.031
	Grain yield	−0.594**	−0.489**	0.646**	−0.594**	−0.412**	0.731**

n = 24; *P < 0.05; **P < 0.01.

## Discussion

### Effects of Applied N and Intercropping on the Growth Characteristics and Yield of Wheat

Ideal plant morphology and canopy structure are not only the basis for high crop yields, but also the goal of super high yield cultivation and breeding (Li et al., 2019). Different nitrogen application levels and planting arrangements significantly affect plant morphology and canopy structure (Li Y. D. et al., 2010). In the present study, plant height, leaf length and width, and the number of leaves in monocropped and IW were found to increase with increasing levels of applied nitrogen, with plant height and numbers of leaves of wheat intercropped with faba beans also increasing significantly (Table 2), consistent with the results of previous studies. The results indicate that the application of nitrogen and intercropping can improve the morphology and canopy structure of wheat which then affects light levels and yield in the wheat canopy.

In agricultural production, nitrogen fertilizer not only affects the characteristics of crop growth, it also influences crop yield. Previous studies have demonstrated that, over a certain range, crop yields increase with an increase in applied nitrogen (Zhu et al., 2017). For example, Wang et al. (2016) demonstrated that the use of nitrogen fertilizer caused grain weight per ear, grain number per spike, 1000-grain weight, and yield of wheat to be significantly different from those of the control, increasing by 42.6, 26.8, 15.4, and 16.0%, respectively. In the present study, the application of nitrogen also had a significant effect on wheat yield (Table 5). Two-year field experiments demonstrated that the application of nitrogen fertilizer increased biomass, yield, and various components of yield in monocropped and IW, but increasing levels of applied nitrogen initially increased spike weight, grain number per spike, 1000-grain weight, biomass, and yield which then decreased, reaching its maximum at the N_2_ level. The same experimental results have been confirmed with Millet (*Panicum miliaceum* L.) (Gong et al., 2017) and barley (*Hordeum vulgare* L.) (Barati et al., 2015). The results demonstrated that an appropriate application of nitrogen fertilizer increased crop yield, but excessive nitrogen fertilizer not only resulted in a lower yield, it also caused nutrient loss due to rapid growth, resulting in unsatisfactory nutrition that preventedoptimal growth and development of the reproductive organs of the crop, causing final crop yield to decrease (Wang et al., 2012). In addition, excessive nitrogen increased crop growth, plant height, numbers of leaves, and other agronomic traits, resulting in crop canopy shading, poor ventilation, and light penetration. Serious outbreaks of wheat powdery mildew, rust, and other diseases have been shown to occur, resulting in a decline in wheat yield (Luo et al., 2021).

Intercropping is not only an effective measure to increase farmland light and heat resource, it also represents the wisdom of traditional agriculture in China. Of the existing intercropping models in China, the model in which a cereal and legume are cropped together not only increases yield and efficiently utilizes resources, the biological nitrogen fixation by legume crops, more importantly, reduces the use of nitrogen fertilizer and so is widely utilized (Zhu et al., 2017). Multiple studies on soybean [*Glycine max* (Linn.) Merr.]/oat (*Avena sativa* L.) (Feng et al., 2015) and millet/mung bean (*Vigna radiata* L. Wilczek) (Gong et al., 2018, 2020) etc., in different intercropping systems have shown that, compared with monocropping, intercropping has clear advantages in terms of increased yield and efficiency. In the present study, the planting arrangement significantly influenced wheat yield (Table 3). Wheat/faba bean intercropping was also advantageous to yield. The yield of wheat intercropped with faba beans increased by 36.34% (2016–2017) and 35.28% (2017–2018) compared with monocropping at the N_0_∼N_3_ levels. LER is an important index for measuring land use efficiency. In the present study, the LER of wheat and faba bean intercropping was 1.32∼1.42 (2016–2017) and 1.29∼1.40 (2017–2018), respectively, demonstrating the clear advantage of intercropping. Feng et al. (2015) demonstrated that the LER of soybean/oat and peanut (*Arachis hypogaea* L.)/oat was 1.41∼1.63 and 1.31∼1.52, respectively, consistent with the results of the present study. In addition, the increase in yield of wheat due to intercropping was 46.83, 38.61, 32.26, and 27.66% (2016–2017), and 47.20, 32.11, 33.82, and 28.00% (2017–2018), at the N_0_–N_3_ levels, respectively. The results indicate that increased levels of nitrogen fertilizer did not increase the advantage of intercropping toward yield, but tended to decrease it. This observation is similar to the results of Wu et al. (2012) in maize (*Zea mays* L.)/potato (*Solanum tuberosum* L.). Although the application of nitrogen increased yields from intercropping, the advantage of intercropping decreased with increasing nitrogen level. This may be due to the two crops sharing a limited space and resources with intercropping, demonstrating interspecies competition, which increased when excessive nitrogen fertilizer was applied, and indicating that the application of nitrogen reduces the advantage of intercropping (Wu et al., 2012). It is also possible that excessive nitrogen seriously reduces the yield of wheat due to diseases, reducing the advantage of intercropping (Zhu et al., 2018).

### Effects of Applied N and Intercropping on the Canopy Light Environment in Wheat and Its Relationship With Yield

Light is the principal source of energy for crop photosynthesis, and so light intensity plays a direct role in determining the growth and development of crops (Al-Dalain, 2009). For the same levels of fertility, soil productivity, and other resources, efficient light energy interception and utilization can be used to improve crop quality, increase efficiency, and provide high yields (Li Y. D. et al., 2010; Raza et al., 2019).

The results demonstrated that the application of nitrogen fertilizer is effective at reducing the light intensity within a crop canopy and increases IPAR (Shi et al., 2014). A study of faba beans by Ma et al. (2019) demonstrated that the addition of nitrogen fertilizer decreased the LT of a faba bean canopy and increased PAR. A study by Wang et al. (2017) demonstrated that increased nitrogen fertilizer resulted in an increased IPAR of the wheat canopy and decreased air temperature, effectively improving the microecological environment of the wheat canopy. In the present study, the application of nitrogen significantly affected canopy LT and IPAR in wheat (Table 3). LT within the canopy of monocropped and IW decreased with increasing nitrogen fertilizer, in the order N_0_ > N_1_ > N_2_ > N_3_. However, IPAR increased initially, then decreased, reaching a maximum at the N_1_ and N_2_ levels, indicating that the IPAR of the wheat canopy could be increased by applying nitrogen fertilizer over a certain range, excessive nitrogen leading to decreased canopy IPAR. A study by Li D. Q. et al. (2010) also demonstrated that the IPAR of rice increased with increasing nitrogen, but decreased when the application of nitrogen exceeded 210 kg/ha. This may be due to wheat leaf diseases, such as powdery mildew and leaf rust, observed following excessive nitrogen application, resulting in the loss of green crop material and the death of a number of leaves, thus reducing IPAR within the canopy (Luo et al., 2021). In addition, excessive nitrogen application leads to excessive numbers of wheat leaves with a canopy that is excessively dense, affecting leaf inclination of the wheat leaves and reducing the interception and utilization of light energy by the canopy (Xu et al., 2010).

Appropriate application of nitrogen can promote crop growth, balance the relationship between canopy LT and IPAR, achieving both light transmittance and effective interception of photosynthetic radiation, allowing the efficient use of light energy, and increasing crop yield (Lei et al., 2016). In the present study, LT and PART in the middle and lower parts of the canopy were significantly negatively correlated with wheat yield and yield factors, while IPAR was significantly positively correlated with wheat yield and yield factors (Table 6). The results demonstrate that low LT, PART, and high IPAR contribute to increased wheat yield, consistent with the results of previous studies (Tang et al., 2012). In the present study, the results of field experiments over 2 years demonstrate that the yield from monocropped and IW was maximum at the N_2_ level, with canopy IPAR almost reaching its maximum at the N_2_ level. Insufficient nitrogen fertilizer reduced the number of tillers and leaves and the leaf area index of the wheat population resulting in serious light leakage in the wheat canopy causing a waste of light energy resources, that did not contribute to enhanced wheat yield. On the other hand, excessive use of nitrogen led to a wheat canopy that was too dense with poor ventilation and light transmission that reduced light levels in the middle and lower parts of the canopy, resulting in reduced photosynthetic productivity that did not contribute to wheat yield (Li Y. D. et al., 2010). Therefore, for the production of wheat, it is important that crops maximize yield by applying a quantity of nitrogen demonstrated to form an efficient canopy structure that makes full use of light energy resources.

Agricultural practices have shown that in intercropping systems, due to differences in plant height and shape, leaf shape, and other characteristics of the two crops, a three-dimensional field canopy structure is often created (Ma et al., 2019). Compared with monocropping, this canopy structure can make full use of the available space and time, improving crop canopy LT and IPAR, and improving crop light energy utilization efficiency, which is important for the regulation of crop growth and development and to obtain a high yield (Wang et al., 2015). The intercropping of pepper and corn was shown to increase their yield by increasing the interception rate and utilization rate of PAR in the canopy (Xu et al., 2010). In the intercropping of millet and mung beans, high numbers of millet plants improved the light distribution and light interception of the crop canopy, thus improving the efficiency of the light energy utilization and enhancing the level of crop productivity (Gong et al., 2018, 2020). In the present study, compared with monocropping, the LT and PART of the wheat canopy at different heights for different N_0_∼N_3_ intercropping was lower than that of monocropping, with IPAR that was higher than that of monocropping (Figures 2–4), confirming previous research results, indicating that decreased canopy LT and increased IPAR contribute to advantages in yield of wheat and faba bean intercropping systems. In the present study, wheat and faba beans were used because the two crops have different growth characteristics, such as plant height and shape, and leaf shape, etc. They form a three-dimensional structure with high and low co-location and density within the field, with leaves of the two crops distributed throughout the available space. The uniform canopy structure of the MW was enhanced, thus LT and PART of the wheat canopy decreased, IPAR increased with appropriate light levels within the canopy, beneficial for increased wheat yield. In addition, wheat and faba beans are two crops with different leaf inclination angles. Wheat leaves generally droop naturally, with a relatively small the leaf inclination angle, while faba bean leaves spread upward, with a leaf inclination angle that is relatively large, therefore, when the two crops are intercropped, it allows the canopy to intercept more photosynthetically active radiation and improve the light energy utilization efficiency, thus laying a good foundation for the high yield of wheat.

## Conclusion

Independent of whether the monocropping or intercropping was used, the application of nitrogen increased wheat plant height, spike leaf length and width, and the number of leaves, yield, and yield factors. Wheat yield was maximum at the N_2_ level. Application of nitrogen resulted in the formation of a canopy with a light environment conducive to increased wheat yield by reducing LT and photosynthetically active radiation transmittance while increasing photosynthetically active radiation interception rate. Compared with monocropping, intercropping increased wheat plant height and leaf number, decreased LT and photosynthetically active radiation transmittance, and increased photosynthetic active radiation interception. This improved canopy structure, optimized the light environment in the canopy, increased light energy utilization efficiency, and significantly increased wheat biomass and yield. Wheat/faba bean intercropping with nitrogen applied at 180 kg/ha are effective methods of obtaining a high yield of wheat in southwest China or in similar environmental conditions.

## Data Availability Statement

The original contributions presented in the study are included in the article/supplementary material, further inquiries can be directed to the corresponding author/s.

## Author Contributions

CL and ZG conceived the original screening, research plans, and designed the experiments. ZG finished original writing this thesis. CL finished re-writing and revising this thesis. KD and YD supervised the experiments and agreed to serve as the author responsible for contact and ensures communication. JX and ZG provided the technical assistance to CL. JX, CL, and ZG analyzed the data. All authors contributed to the article and approved the submitted version.

## Conflict of Interest

The authors declare that the research was conducted in the absence of any commercial or financial relationships that could be construed as a potential conflict of interest.

## Publisher’s Note

All claims expressed in this article are solely those of the authors and do not necessarily represent those of their affiliated organizations, or those of the publisher, the editors and the reviewers. Any product that may be evaluated in this article, or claim that may be made by its manufacturer, is not guaranteed or endorsed by the publisher.

## Abbreviations

LT: canopy light transmittance
PART: canopy photosynthetically active radiation transmittance
IPAR: canopy photosynthetically active radiation interception rate
MW: monocropped wheat
IW: intercropped wheat.
